# Safe Exercise at Home Website: Keeping Older People Active during COVID-19

**DOI:** 10.1093/geroni/igaa057.3497

**Published:** 2020-12-16

**Authors:** Anne-Marie Hill, Cathy Said, Cathie Sherrington, Keith Hill, Frances Batchelor, Courtney West, Shylie Mackintosh, Michele Callisaya

**Affiliations:** 1 Curtin University, Perth, Western Australia, Australia; 2 University of Melbourne, Melbourne, Victoria, Australia; 3 University of Sydney, Sydney, New South Wales, Australia; 4 Monash University, Melbourne, Victoria, Australia; 5 The University of Melbourne, Melbourne, Victoria, Australia; 6 University of South Australia, Adelaide, South Australia, Australia; 7 Monash University, Frankston, Australia

## Abstract

Background Physical distancing restrictions, including recommendations to ‘stay home’ during the COVID-19 pandemic has restricted exercise opportunities for older adults. Our group of academic and clinical physiotherapists and a communications expert identified the need to support safe exercise at home to minimise the impact of these restrictions. The project aimed to develop an online, publicly available resource to support older adults to exercise at home. We met virtually and: a) developed home exercise programs for people at three levels of functional ability; b) developed simple advice about exercising safely, exercise intensity, and staying motivated; and c) reviewed and selected online exercise programs and resources for consumers and health professionals to access. Examples of older adults keeping active during the pandemic were sourced to provide motivation. Website content was made available to download and print to increase accessibility. Modifications were made after consumer and international advisor feedback, the website was endorsed by the Australian Physiotherapy Association. Content is updated as restrictions are modified and in response to feedback received. Google Analytics was used to evaluate website usage. The website was launched on 5 May 2020, 35 days after the group’s initial meeting. In the first 9 weeks of website availability 20,608 users accessed the website, with 14.6% being returning users. There were 27,513 sessions and 74,927 page views. Most users were from Australia (80.74%), followed by Denmark (5.15%) and USA (3.85%) We present this example of the benefits of time-critical collaboration to facilitate rapid translation of evidence into practice.

